# The Relationship Between Familial Functioning and Social Media Use Among Children with Depression and Attention Deficit Hyperactivity Disorder: A Comparative Study with Healthy Controls

**DOI:** 10.3390/children12070906

**Published:** 2025-07-09

**Authors:** Mutlu Muhammed Özbek, Doğa Sevinçok, Emre Mısır

**Affiliations:** 1 Department of Child and Adolescent Psychiatry, Yalova University, Yalova 77200, Türkiye; 2 Department of Child and Adolescent Psychiatry, İstinye University, İstanbul 34396, Türkiye; doga.sevincok@istinye.edu.tr; 3 Department of Psychiatry, Başkent University, Ankara 06790, Türkiye; emre.misir@baskent.edu.tr

**Keywords:** social media, adolescent behavior, internalizing symptoms, family relations, parent–child relations

## Abstract

Objective: This study aimed to investigate the relationship between social media use (SMU) in children diagnosed with major depression (MD) or attention deficit hyperactivity disorder (ADHD) and various psychosocial factors, including familial functioning, parental SMU, and parent-reported internalizing and externalizing symptoms. A healthy control group was included for comparison. Methods: The study included 121 children and adolescents aged 10–18 years (36 with MD, 41 with ADHD, and 44 healthy controls). The Social Media Addiction Scale—Short Form (SMDS) was administered to all participants, while mothers completed the McMaster Family Assessment Scale (FAS), the Social Media Addiction Scale—Adult Form (SMAS-AF), and the Child Behavior Checklist (CBCL). Psychiatric diagnoses were made using the K-SADS-PL DSM-5-T. Correlation and linear regression analyses were used to assess associations among variables. Results: SMU scores were significantly higher in the ADHD group compared to both the depression and control groups. Parental SMU was also higher in the ADHD group. In the depression group, child SMU was significantly associated with internalizing symptoms and impaired family communication. In the ADHD group, child SMU was predicted by poor family problem-solving and communication. Regression analyses showed that internalizing symptoms and family communication predicted SMU in the depression group (R^2^ = 0.335), while family problem-solving and communication predicted SMU in the ADHD group (R^2^ = 0.709). Conclusion: The findings suggest that social media use in children with depression and ADHD is associated with different psychosocial factors. While internalizing symptoms and family communication are more prominent in depressed children, family functioning—particularly problem-solving and communication—plays a larger role in children with ADHD. These results emphasize the need for targeted family-based interventions to mitigate problematic SMU in clinical populations.

## 1. Introduction

Social media is defined as a computerized technology that enables the sharing of thoughts, ideas, and information with others using mobile and web-based applications [1]. Social media use (SMU) is rapidly increasing across age groups, especially among adolescents [2]. In the literature, SMU is referred to as extreme SMU, problematic SMU, social network aggregation, and social network disorder [3,4,5]. Although symptoms related to addiction are prominent, DSM 5 does not include a diagnosis for social media addiction. The most appropriate addiction diagnosis for this situation in DSM 5 is internet gaming disorder [6]. The recent literature increasingly points to a connection between SMU and mental health outcomes in adolescents [7]. A meta-analysis of studies involving adolescents showed a statistically significant correlation between depressive symptoms and problematic SMU [8]. It has been reported that excessive SMU is associated with internalization, externalization symptoms, and depressive mood, and the link between depression and problematic SMU may be bidirectional [9]. As the time spent on social media increases, people will be more depressed, which will be affected by negative reactions [10]. The relationship between SMU and depression may be significantly mediated by personality traits such as jealousy, rumination, extraversion, and cyber-bullying, perceived social support, negative comparison in social settings, and distorted body image with increased physical anxiety [11,12,13,14,15]. One factor that increases the likelihood of behavioral addiction is attention deficit hyperactivity disorder (ADHD), a neuropsychiatric disorder characterized by inattention and/or hyperactivity-impulsivity that affects an individual’s functioning in social, work, and school domains [6]. Individuals with attention deficit hyperactivity disorder may develop behavioral or substance abuse to cope with distressing behaviors and thoughts [16]. Several studies suggest significant links between ADHD and internet-related addictive behaviors, including video gaming and SMU in adolescence [17,18]. Individuals with ADHD tend to engage more frequently with digital media, particularly highly stimulating and interactive content such as video games and social media platforms [19]. Environmental factors such as parental supervision and the context in which digital media is used play a critical role in moderating its effects on ADHD symptoms [20]. The increasing social difficulties, especially during adolescence, may require adolescents with ADHD to seek new ways to establish social relationships [21,22]. Impulsivity, one of the core symptoms of ADHD, is thought to result from rapid and unplanned responses to immediate feedback in children and adolescents, and immediate social feedback may increase the adolescent’s need for reward, leading to problematic SMU [23].

The family is the primary source of meeting basic social and psychological needs throughout the child’s developmental process [24]. Family relationships play an important role in shaping children’s development, providing a foundation for socialization, emotional support, and identity formation. Healthy family relationships are characterized by open communication, mutual respect, and a sense of belonging, and provide a secure base from which adolescents can explore their identities and relationships with others [25]. It has been reported that problems between mothers and adolescents affect the time spent by the adolescent and the subsequent establishment of online friendships [26]. Positive family functioning, as indicated by strong communication and cohesion in the family, has been found to be a protective factor against internet addiction [27]. The deterioration in family functioning may cause the child to become overly focused on the digital world, develop a SMU disorder, and become more prone to problematic online behaviors. Conflictive parent–child relationships, such as poor communication and frequent serious arguments, are important environmental factors in developing SMU in adolescence [28,29,30]. Parents’ media use has been found to be associated with impaired parent–child communication, characterized by slower, less attentive, and more passive interaction with their children [31]. Severe SMU in parents has been associated with problematic SMU in their children. Increased parental internet use has been observed to lead to less parent–child interaction and less sensitivity to the child’s wishes [32]. Although previous studies have found associations between problematic SMU and depression and ADHD in children, the impact of family functionality and parents’ SMU on problematic SMU in these groups has not been adequately examined. Therefore, this study focused on determining the possible relationships between SMU in children with ADHD and depression in terms of family functionality in various domains. We examined whether the relationships between family functionality, parental SMU, parent-reported psychopathology of children, and children’s SMU differed between children diagnosed with ADHD and those diagnosed with depression. Accordingly, our first hypothesis is that the levels of SMU among children and their parents will significantly differ across the depression, ADHD, and healthy control groups. The second hypothesis posits that levels of family functioning will show significant differences among these three groups. The third hypothesis suggests that children’s SMU will be significantly associated with both family functioning and parental social media use. The fourth hypothesis proposes that SMU will be significantly associated with internalizing symptoms in children diagnosed with depression, whereas it will be significantly associated with externalizing symptoms in children diagnosed with ADHD.

## 2. Methods

Children and adolescents aged 10–18 years, diagnosed with major depression or ADHD, who applied to the Child and Adolescent Psychiatry outpatient clinic of the Training and Research Hospital between 14 February 2025 and 1 May 2025, were included in this cross-sectional study. The healthy control group was determined among children and adolescents who applied to the general pediatric outpatient clinic for routine control. The study was approved by the Institutional Review Board of University (2025/24). After detailed information about the study was provided by the authors, written informed consent was obtained from the legal guardians of the participating children and adolescents. All participants were assessed by an experienced senior child and adolescent psychiatrist (M.M.O). In order to determine MD, ADHD, and comorbid psychiatric disorders, the Schedule for Affective Disorders and Schizophrenia for School Age Children Present and Lifetime Version DSM-5-Turkish Version (K SADS PL DSM-5-T eklenecek) was applied to three groups. Exclusion criteria for patient groups were bipolar disorder, psychotic disorders, autism spectrum disorders, substance use disorders, intellectual disabilities, and any neurological disease. There were no lifetime diagnoses of psychiatric or neurological disorders in the healthy control group. Therefore, 36 children with MD, 41 children with ADHD, and 44 healthy controls were included in our sample for data analysis.

## 3. Instruments

Information about the participants and their mothers such as age, gender, education level, marital status, and type of delivery was collected through a sociodemographic form. The Short Form of the Social Media Addiction Scale For Adolescents was administered to all children. The mothers completed the McMaster Family Assessment Scale (FAS), Social Media Addiction Scale—Adult form, and Child Behavior Check List For Ages/4–18.

### 3.1. K-SADS-PL DSM-5-T

Psychiatric assessment was performed using the Turkish version of the (K-SADS-PL), based on the Diagnostic and Statistical Manual of Mental Disorders, Fifth Edition, diagnostic criteria [33]. K-SADS-PL DSM-5-T consists of three sections [33]. The first section involves an unstructured interview to assess developmental history, health status, and general functioning. The second section evaluates specific symptoms through screening questions in terms of both current and past symptoms. Moreover, the third section aims to confirm the diagnosis, which is significant in the screening section.

### 3.2. The Social Media Addiction Scale-Short Form for Adolescents (SMAS-S)

The nine-item short form of the SMAS-S, developed by Van den Eijnden et al. (2016) to measure adolescents’ addiction levels to social media, was used in our study [5]. The criteria under the title Internet Gambling Disorders in DSM-V were taken as a basis and the item pool was created according to a total of nine criteria (preoccupation, endurance, deprivation, insistence, escape, problems, deception, displacement, conflict). The scale was self-reported by the child participants and aimed to capture the subjective severity of problematic social media use from the adolescent’s perspective. A higher score indicates a higher risk of social media addiction. A Turkish validity and reliability study was conducted by [34].

### 3.3. McMaster Family Assessment Scale (FAS)

The Turkish version of the FAS, which evaluates functions according to families’ perceptions, was completed by mothers [35]. FAS was developed by applying the McMaster Model of Family Functioning clinically to families [36]. The scale, which consists of a total of 60 items, has subscales of problem solving, communication, roles, emotional responsiveness, emotional involvement, behavioral control, and general functionality. Each of these subscales is scored on a 4-point Likert scale (1 = Strongly Disagree, 4 = Strongly Agree). Higher scores generally indicate greater functional impairment. While helping to evaluate the overall functioning of the family as a whole, it provides a more systemic perspective by assessing both structural and functional family dynamics.

### 3.4. Child Behavior Check List (CBCL) for Ages 6–18

The Turkish version of the CBCL-6–18 was administered to mothers [37]. Child Behavior Checklist/6–18 is a 113-item measure developed to assess behavioral and emotional problems in children and adolescents aged 6–18 years. The internalization scale assesses withdrawn, somatic complaints and anxious/depressive symptoms. The externalizing scale assesses symptoms of aggressive and delinquent behaviors. It is used to assess children’s emotional and behavioral symptoms based on parental reports and contributes to a comprehensive, multidimensional evaluation of the child’s mental health.

### 3.5. Social Media Addiction Scale-Adult Form (SMAS-A)

This is a measurement tool used to determine the social media addiction of adults between the ages of 18–60. This scale, consisting of 20 sub-items, was created by [38]. The items assess individuals’ levels of preoccupation with social media, increased use (tolerance), withdrawal symptoms, usage for escape purposes, and the emergence of academic or social problems—reflecting patterns of behavior similar to addiction. Each item is structured to evaluate the extent to which these patterns apply to the individual’s social media use habits. Cronbach Alpha internal consistency coefficient was found as 0.94 for the overall scale, 0.92 for virtual tolerance, and 0.91 for virtual communication subdimensions.

## 4. Statistical Analysis

In this study, the Statistical Package for Social Sciences (SPSS), version 25 (IBM SPSS Statistics for Windows), was used to perform statistical analyses. Descriptive statistics for continuous variables are shown as Mean (m) and Standard Deviation (SD) for each group. Additionally, number (n) and percentage (%) values were shown for categorical variables. Assumptions of normality in each group were tested using histogram plots as well as Skewness and Kurtosis values. Corrective analyses were performed for variables that did not conform to normal distribution, and extreme values were removed from the data. With this method, all data was brought into compliance with normal distribution for each group. One-way ANOVA analysis was applied to determine whether continuous variables differed between groups. When significant differences were detected between groups, post hoc analyses were applied to examine which groups caused the significant differences. LSD method was used in post hoc analyses. Differences in categorical variables between groups were examined with Chi-Square test. After determining the differences between groups, Pearson Correlation Test was performed to determine the relationship between clinical variables separately in both the depression group and ADHD group. Multicollinearity was not detected in either the ADHD or depression groups. Therefore, regression analyses were conducted to identify the factors associated with SMU. Additionally, VIF values were reported to assess multicollinearity. No multicollinearity was observed, including at the subscale level. Then, Linear Regression Analysis was applied with Enter method to predict SMU in children; variables included in the model as independent variables were determined according to the correlation analysis results, and separate models were created for depression and ADHD research groups. However, the FAS-Affective Involvement variable was not included in the regression analysis in the ADHD group due to a multicollinearity problem. In all analyses, a *p* < 0.05 value was accepted as statistically significant.

## 5. Results

### 5.1. Group Comparisons

The sociodemographic and clinical characteristics of the participants are shown in Table 1 and Table 2. The mean ages of the participants did not differ significantly between the groups (*p* = 0.060). However, it was found that the mean ages of the participants’ mothers and fathers differed significantly between the groups, and the mother’s age was lower in the ADHD group than in the control group (*p* = 0.014), and the father’s age was significantly lower in the depression group than in the control group (*p* = 0.031).

The comparison revealed a statistically significant difference in gender distribution across the groups (*p* < 0.001). The cases in the depression group were predominantly female (72.2%), the ADHD group was predominantly male (85.4%), and 56.8% of the participants in the control group were female. A comparison of delivery types showed a statistically significant lower frequency of C/S in the depression group relative to controls (*p* = 0.007) (Table 1).

As indicated in Table 2, child social media use scores measured with SMDS were found to be significantly higher in the ADHD group than in both groups (*p* < 0.001). Parent social media use scores measured with SMAS-AF were also found to be higher in parents of children with ADHD compared to the other groups (*p* < 0.001). No statistically significant variation in SMU was observed between individuals in the depression and control groups. A significant difference was found between the groups in the FAS Communication (*p* = 0.004), Roles (*p* = 0.005), Affective Involvement (*p* = 0.020), and General Functioning (*p* = 0.002) subscale scores. Post hoc analyses showed that Communication subscale scores were significantly higher in both the depression and ADHD groups than in the control group, but there was no significant difference between the ADHD and depression groups. Roles and Emotional Involvement subscale scores were significantly higher in the ADHD group than in both groups. Scores on the General Functioning subscale were found to be notably higher in participants with ADHD than in those in the control group. In addition, CBCL-Internalization scores also showed significant differences (*p* < 0.001), and in post hoc analyses, significantly higher Internalization scores were found in the depression and ADHD groups compared to the control group. CBCL-Externalization was found to be higher in the ADHD group compared to the other groups (*p* < 0.001) (Table 2).

### 5.2. Correlation Analysis

Correlation analyses were conducted to understand the relationships between clinical variables within the groups. In the depression group, SMDS scores indicating social media use in children were found to be significantly correlated with FAS-Problem Solving (r = 0.367, *p* = 0.028), FAS-Communication (r = 0.439, *p* = 0.007) and CBCL-Internalization (r = 0.343, *p* = 0.041). SMAS-AF scores showing parental social media use were found to be correlated with FAS-Communication (r = 0.631, *p* < 0.001), FAS-Roles (r = 0.428, *p* = 0.009), FAS-Affective Responsiveness (r = 0.559, *p* < 0.001), FAS-Affective Involvement (r = 0.644, *p* < 0.001), and CBCL-Internalization (r = 0.568, *p* < 0.001). In addition, CBCL-Internalization scores were correlated only with Affective Involvement among the FAS subscales (r = 0.476, *p* = 0.003) (Table 3). To ensure transparency, the full correlation analysis for the control group has been provided as Supplementary Table S1.

SMDS scores in the ADHD group were found to be associated with FAS-Problem Solving (r = 0.549, *p* < 0.001), FAS-Communication (r = 0.523, *p* < 0.001), FAS-Affective Involement (r = 0.338, *p* = 0.001), FAS-General Functioning (r = 0.372, *p* = 0.017), and CBCL-Externalization (r = 0.360, *p* = 0.021). SMAS-AF scores were found to be associated with FAS-Problem Solving (r = 0.409, *p* = 0.008), FAS-Affective Involvement (r = −0.320, *p* = 0.041), CBCL-Internalization (r = 0.619, *p* < 0.001), and CBCL-Externalization (r = 0.347, *p* = 0.026) (Table 4).

### 5.3. Regression Analysis

In our study, linear regression analyses were applied with the Enter method to determine the variables considered to influence SMU in children. The results of the linear regression analysis performed in the depression group are shown in Table 5. In the model where SMDS was included as the dependent variable, CBCL-Internalization, FAS-Problem Solving, and FAS-Communication were included as independent variables. The regression model was found to be statistically significant (F = 5.364, *p* < 0.05, R2 = 0.335, Dubin–Watson = 2.081, max VIF value = 2.122). The model explains 35% of the variance in the dependent variable. According to the regression analysis results, it was concluded that the variables CBCL-Internalization (B = 0.526, β = 0.394, *p* = 0.017) and FAS-Communication (B = 1.634, β = 0.511, *p* = 0.018) had a significant effect on SMDS independent from other variables. The results of the linear regression analysis performed in the ADHD group are shown in Table 6. In the model where SMDS is included as the dependent variable, CBCL-Externalization, FAS-Problem Solving, and FAS-Communication are included as independent variables. The regression model was found to be statistically significant (F = 30.112, *p* < 0.05, R2 = 0.709, Dubin–Watson = 1.717, max VIF value = 1.351). The model explains 70% of the variance in the dependent variable. According to the regression analysis results, it was concluded that FAS-Problem Solving (B = 2.659, β = 0.691, *p* < 0.001) and FAS-Communication (B = 1.910, β = 0.681, *p* < 0.001) variables have a significant effect on SMDS independent of other variables.

## 6. Discussion

The aim of this study was to determine the relationship between SMU in children diagnosed with depression and ADHD and family functioning, parental SMU, and parent-reported internalizing and externalizing symptoms. In our study, some sociodemographic variables showed significant differences between groups. In particular, gender distribution (with a higher proportion of males in the ADHD group and a higher proportion of females in the depression group) and parental age averages differed significantly across groups. However, it should be noted that these differences largely align with clinically expected patterns. Indeed, the higher prevalence of ADHD in males and depression in females is a well-documented finding in the literature [39,40]. Therefore, the distribution in our sample is considered consistent with the clinical characteristics of the study population, and it is not believed to undermine the validity of the results obtained.

According to the results, children with ADHD had higher child and mother SMU scores than children with depression and healthy controls. Apart from drug treatments, some therapeutic interventions, such as cognitive behavioral therapy (CBT) or group counseling, can limit social media use and improve well-being by helping individuals regulate their social media use [41,42]. We also found that SMU in children with depression was significantly associated with the severity of children’s internalizing symptoms and family communication problems. On the other hand, SMU severity in children with ADHD was predicted by family problem-solving and communication problems. Therefore, we suggest that SMU severity in children with depression and ADHD has different associations with child- and maternal-related factors. Studies suggest a strong association between adolescent depression and excessive SMU [43,44]. Furthermore, a positive association has been found between heavy SMU (2 h or more per day) and internalizing disorders (depression and anxiety) especially in younger adolescents [43]. Various evidence-based counseling approaches such as Cognitive Behavioral Therapy, Motivational Interviewing, and Problem Solving are used to cope with depression and anxiety in young adolescents, understand the positive and negative uses of SMU, and encourage them to refrain from using social media [45].

In adolescents, problematic SMU may develop as a way to regulate emotions, cope with feelings of alienation and neglect, and reduce stress [46]. Other studies have shown that adolescents engage in pathological internet use to manage symptoms of anxiety, depressed mood, worry, and to increase a sense of acceptance and belonging [47]. Our findings consistently showed that SMU was significantly associated with parent-reported internalizing symptoms such as depression and anxiety in children with depression, but not in children with ADHD. An adolescent who exhibits internalizing symptoms such as anxiety, depression, negative emotionality toward oneself, repetitive thought patterns, and social withdrawal may be affected differently by social media than an adolescent with an externalizing condition such as impulsivity, risk taking, and lack of inhibition. Because depressed adolescents with internalizing conditions spend more time on social media and engage in more social comparisons, feedback on social media may have a greater impact on mood [39]. One of the most important findings of this study is that communication problems in families were significantly associated with SMU in children with both depression and ADHD. Additionally, problems in parents’ problem-solving skills appear to affect the severity of SMU in children with ADHD. Armsden and Greenberg identified communication as an aspect of the parental attachment construct that would help create a strong emotional bond between parents and children. Adolescents who display problematic SMU symptoms may engage in unhealthy family functioning, which may lead to increased conflict, poor communication, and low levels of cohesion in the family [48,49]. The time parents spend with children and the relationship between parents and children are negatively associated with adolescents’ addiction [50]. In families of children with ADHD, parent–child relationships are more negative and conflictual compared to families without ADHD [51]. Adolescents may use media to escape from negativity in the family environment, and to transfer negative emotions to the online world [52]. Dysfunctional families are those characterized by rigid boundaries, poor communication, and ineffective problem-solving strategies [53]. A dysfunctional family is one that experiences emotional turmoil, conflict, and a lack of ability to work together as a cohesive unit due to poor or unhealthy relationships, communication, and interactions. Unresolved conflicts, distorted family roles, poor communication styles, emotional dysphoria, and unhealthy coping strategies such as dependency are potential signs of dysfunctional families. It has been suggested that children in dysfunctional families often experience problems such as parental inconsistencies, conflicting messages, hidden emotions, hidden information, shame, uncertainty, and insecurity, which can lead to problems in later years. A significant number of social scientific studies demonstrate the connections between faith and family life by identifying key dimensions that contribute to understanding how shared faith affects family dynamics, such as religious community, religious practices, and religious beliefs. Findings suggest that family prayer and shared faith can strengthen marriages and provide valuable coping resources during times of adversity [54].

Children with ADHD and their parents have demonstrated conflictual parent–child interaction patterns and dysfunctional parenting styles in observational studies [55,56]. Children with ADHD have an impact on family functioning because their parents have difficulty controlling and disciplining them [40]. Some previous studies have reported that children with ADHD are less compliant and more negative, with more directive and negative parents. Increased symptomology has been associated with less warmth and involvement during problem solving. Additionally, lack of parental supervision may amplify these effects by allowing for erratic and excessive media consumption [57].

Contrary to our expectations, in this study, although parental SMU was higher in children with ADHD compared to depressed children and healthy controls, mothers’ SMU was not associated with SMU in children with ADHD in this study. However, previous studies have indicated that parents’ SMU significantly influences their children’s behaviors. A previous study observed that children’s mental health and parents’ internet use mediated the relationship between parental mental health and children’s internet addiction [58]. Parental pathological internet use can contribute to inconsistent or negative parenting practices, such as poor communication, which increases parent–adolescent conflict [59]. These findings align with research emphasizing poor communication and conflict as possible mediating pathways linking parent and adolescent behaviors [60]. We recognize several limitations inherent in our study. First, due to its cross-sectional design, the data do not allow for any causal interpretations or directional conclusions. Moreover, the relatively limited sample size across different groups warrants caution when interpreting findings, particularly in relation to interaction effects. Our data collection also relied on self-reported measures, which, while valuable in capturing subjective experiences such as online social comparison, are inherently limited in objectivity and reliability. To better understand the temporal and causal links between various SMU patterns and mental health outcomes, longitudinal research is essential. Another noteworthy limitation is the absence of a specific assessment of the relationship between SMU and the severity of depression and ADHD symptoms.

In conclusion, our results demonstrated that SMU in children with depression and ADHD were somewhat associated with different factors. SMU in children with ADHD was significantly predicted by problems in family problem-solving and communication, whereas SMU in depressed adolescents was significantly associated with internalization symptoms and problems in family communication. 

The present findings, indicating that problematic social media use (SMU) in children with attention deficit hyperactivity disorder (ADHD) and depression is associated with distinct familial and psychological factors, may suggest the need for more targeted, preventive, and family-centered approaches in future health policy planning. In the case of children with ADHD, it may be advantageous for policy frameworks to consider incorporating parent-focused intervention programs, particularly those aimed at enhancing family communication and problem-solving skills—within existing child and adolescent mental health services. For children with depression, the implementation of routine screening procedures for internalizing symptoms and assessments of family relational dynamics in primary care or school-based mental health settings could potentially facilitate earlier identification of individuals at heightened risk. Moreover, national adolescent digital media use guidelines might benefit from accounting for differing risk profiles across clinical populations. In this context, promoting family-based digital literacy education could serve as a protective strategy to mitigate the possible adverse effects of dysfunctional parenting practices and emotional neglect.

## Figures and Tables

**Table 1:** Sociodemographic and clinical variables.

	Depression (a) n = 36		ADHD (b) n = 41		Control (c) n = 44		Statistical Analyses		
Variables	m	SD	m	SD	m	SD	F *	*p*	Post Hoc
Age	13.38	1.33	12.31	1.34	12.84	2.71	2.88	0.060	
Mother’s Age	39.83	4.43	39.58	6.06	42.77	5.64	4.43	0.014	a = b, a = c, b < c
Father’s Age	44.08	5.40	44.75	5.66	47.40	6.62	3.59	0.031	a = b, a < c, b = c
Birth Weight (kg)	3317.36	679.43	3314.63	355.71	3134.72	493.84	1.71	0.185	
First Speech (mo)	16.19	7.77	17.14	6.24	15.06	6.07	1.03	0.359	
	n	%	n	%	n	%	x^2^ **	*p*	
Gender Boy Girl	10 26	27.8 72.2	35 6	85.4 14.6	19 25	43.2 56.8	28.13	<0.001	a ≠ b a = c b ≠ c
Marital Status Married Divorced	34 2	94.4 5.6	34 7	82.9 17.1	41 3	93.2 6.8	3.59	0.166	
Problematic Pregnancy Yes No	7 29	19.4 80.6	4 37	9.8 90.2	8 36	18.2 81.8	1.68	0.432	
Type of Delivery Normal C/S	28 8	77.8 22.2	25 16	61.0 39.0	19 25	43.2 56.8	9.89	0.007	a = b a ≠ c b = c

* One-Way ANOVA ** Chi-Square Test Mean (m), Standard Deviation (SD), Number (n) and Percentage (%) were reported.

**Table 2:** Comparison of clinical variables between study and control groups.

	Depression (a) n = 36		ADHD (b) n = 41		Control (c) n = 44		Statistical Analyses		
Variables	m	SD	m	SD	m	SD	F *	*p*	Post hoc
SMDS	18.25	10.28	38.39	16.27	15.50	12.71	35.70	<0.001	b > a = c
SMAS-AF	41.13	16.64	52.09	20.44	37.25	11.77	9.01	<0.001	b > a = c
FAS-Problem Solving	12.25	3.59	10.75	4.22	11.17	3.09	1.68	0.191	
FAS-Communication	19.55	3.21	19.58	5.80	16.72	3.86	5.70	0.004	a = b > c
FAS-Roles	23.02	3.70	26.68	7.09	23.21	5.09	5.60	0.005	b > a = c
FAS-Affective Responsiveness	11.91	3.65	12.02	4.67	10.22	3.40	2.74	0.068	
FAS-Affective Involvement	16.25	3.32	18.02	3.11	16.24	3.31	4.05	0.020	b > a = c
FAS-Behavioral Control	17.88	3.57	19.04	3.75	18.43	3.35	1.02	0.363	
FAS-General Functioning	21.55	5.38	24.78	7.71	19.97	5.27	6.46	0.002	a = c, a = b, b > c
CBCL-Internalization	22.33	7.69	21.48	6.22	9.22	6.58	48.38	<0.001	a = b > c
CBCL-Externalization	8.38	6.39	23.60	16.96	6.77	5.96	28.89	<0.001	b > a = c

* One-Way ANOVA Mean (m) and Standard Deviation (SD) were reported; SMDS: Social Media Disorder Scale; SMAS-AF: Social Media Addiction Scale-Adult Form; FAS: Family Assessment Scale; CBCL: Child Behavior Checklist.

**Table 3:** Correlations between clinical variables in children with depression (n = 36).

	1	2	3	4	5	6	7	8	9	10
1. SMDS	-									
2. SMAS-AF-Total	−0.022	-								
3. FAS-Problem Solving	0.367 *	0.228	-							
4. FAS-Communication	0.439 *	0.631 *	0.671 *	-						
5. FAS-Roles	0.150	0.428 *	0.621 *	0.676 *	-					
6. FAS-Affective Responsiveness	0.201	0.559 *	0.436 *	0.683 *	0.676 *	-				
7. FAS-Affective Involvement	−0.084	0.644 *	0.112	0.446 *	0.261	0.530 *	-			
8. FAS-Behavioral Control	−0.034	0.031	0.473 *	0.105	0.382 *	0.270	0.094	-		
9. FAS-General Functioning	0.304	0.346	0.754 *	0.736 *	0.629 *	0.477 *	0.442 *	0.312	-	
10. CBCL-Internalization	0.343 *	0.568 *	−0.233	0.068	0.081	0.224	0.476 *	0.023	0.057	-
11. CBCL-Externalization	0.153	0.087	−0.160	−0.043	−0.021	0.137	0.241	0.319	0.096	0.324

Pearson Correlation Analysis: Pearson correlation coefficients (r values) were reported. * *p* < 0.05; SMDS: Social Media Disorder Scale; SMAS-AF: Social Media Addiction Scale-Adult Form; FAS: Family Assessment Scale; CBCL: Child Behavior Checklist.

**Table 4:** Correlations between clinical variables in children with ADHD (n = 41).

	1	2	3	4	5	6	7	8	9	10
1. SMDS	-									
2. SMAS-AF-Total	0.064	-								
3. FAS-Problem Solving	0.549 *	0.409 *	-							
4. FAS-Communication	0.523 *	−0.033	−0.186	-						
5. FAS-Roles	0.116	0.121	−0.366 *	0.717 *	-					
6. FAS-Affective Responsiveness	0.300	−0.177	−0.362 *	0.916 *	0.809 *	-				
7. FAS-Affective Involvement	0.338 *	−0.320 *	−0.347 *	0.898 *	0.633 *	0.886 *	-			
8. FAS-Behavioral Control	0.248	0.151	−0.054	0.709 *	0.824 *	0.694 *	0.706 *	-		
9. FAS-General Functioning	0.372 *	0.244	−0.111	0.776 *	0.941 *	0.778 *	0.582 *	0.797 *	-	
10. CBCL-Internalization	0.186	0.619 *	0.214	0.227	0.182	0.178	−0.010	0.095	0.283	-
11. CBCL-Externalization	0.360 *	0.347 *	0.222	0.410 *	0.595 *	0.476 *	0.152	0.456 *	0.728 *	0.521 *

Pearson Correlation Analysis: Pearson correlation coefficients (r values) were reported. * *p* < 0.05; SMDS: Social Media Disorder Scale; SMAS-AF: Social Media Addiction Scale-Adult Form; FAS: Family Assessment Scale; CBCL: Child Behavior Checklist.

**Table 5:** The results of the linear regression analysis (Enter method) conducted to determine variables predicting Social Media Disorder Scores in children with Depression (n = 36).

Dependent Variable: SMDS	B	SE	β	t	*p*	VIF
Constant	0.428	9.849	-	0.043	0.966	-
CBCL-Internalization	0.526	0.209	0.394	2.522	0.017	1.172
FAS-Problem Solving	−0.193	0.601	−0.068	−0.322	0.750	2.122
FAS-Communication	1.634	0.654	0.511	2.498	0.018	2.016

One-Way ANOVA Mean (m) and Standard Deviation (SD) were reported; VIF: Variance Inflation Factor; R^2^ = 0.335, adj. R^2^ = 0.272, F = 5.364, Dubin–Watson = 2.081; SMDS: Social Media Disorder Scale; FAS: Family Assessment Scale; CBCL: Child Behavior Checklist.

**Table 6:** The results of the linear regression analysis (Enter method) conducted to determine variables predicting Social Media Disorder Scores in children with ADHD (n = 41).

Dependent Variable: SMDS	B	SE	β	t	*p*	VIF
Constant	−25.987	7.072	-	−3.674	0.001	-
CBCL-Externalization	−0.069	0.099	−0.072	−0.701	0.487	1.351
FAS-Problem Solving	2.659	0.368	0.691	7.229	<0.001	1.164
FAS-Communication	1.910	0.287	0.681	6.660	<0.001	1.331

One-Way ANOVA Mean (m) and Standard Deviation (SD) were reported; VIF: Variance Inflation Factor; R^2^ = 0.709, adj. R^2^ = 0.686, F = 30.112, Dubin–Watson = 1.717; SMDS: Social Media Disorder Scale; FAS: Family Assessment Scale; CBCL: Child Behavior Checklist.

## Data Availability

The data presented in this study are available on request from the corresponding author. The data are not publicly available due to privacy reasons.

## Supplementary Materials

The following supporting information can be downloaded at: https://www.mdpi.com/article/10.3390/children12070906/s1, Table S1: Correlations Between Clinical Variables in control group (n = 44).
