# Supplementary material for: The Relationship Between Familial Functioning and Social Media Use Among Children with Depression and Attention Deficit Hyperactivity Disorder: A Comparative Study with Healthy Controls
Source: Children (Basel). 2025 Jul 9;12(7):906. doi: 10.3390/children12070906 (PMC12294095; doi:10.3390/children12070906)
Supplement: Supplementary file 1 [file children-12-00906-s001.zip › children-3737734-supplementary.pdf]

Table S1. Correlations Between Clinical Variables in control group (n=44).

	1	2	3	4	5	6	7	8	9	10
**1. SMDS**	-									
**2. SMAS-AF-Total**	0.152	-								
**3. FAS-Problem Solving**	0.033	0.352*	-							
**4. FAS-Communication**	0.063	0.142	0.491*	-						
**5. FAS-Roles**	-0.022	0.144	0.433*	0.206	-					
**6. FAS-Affective Responsiveness**	-0.025	0.033	0.329*	0.518*	0.283	-				
**7. FAS-Affective Involement**	0.081	0.207	0.307*	0.393*	0.314*	0.463*	-			
**8. FAS-Behavioral Control**	-0.008	0.226	0.397*	0.259	0.385*	0.067	-0.023	-		
**9. FAS-General Functioning**	0.047	0.161	0.628	0.689*	0.523*	0.661*	0.592*	0.253	-	
**10. CBCL-Internalization**	0.252	0.190	0.146	0.105	0.155	-0.150	0.204	-0.135	0.201	-
**11. CBCL-Externalization**	0.186	0.156	0.281	0.270	0.169	0.125	0.136	-0.090	0.339*	0.682*

Pearson Correlation Analysis Pearson correlation coefficients (r values) were reported. *$p<0.05$; SMDS: Social Media Disorder Scale; SMAS-AF: Social Media Addiction Scale-Adult Form; FAS: Family Assessment Scale; CBCL: Child Behavior Checklist.
